# Green Synthesis of Selenium Nanoparticles From Clove and Their Toxicity Effect and Anti-angiogenic, Antibacterial and Antioxidant Potential

**DOI:** 10.7759/cureus.55605

**Published:** 2024-03-05

**Authors:** Archana Behera, Mukesh Kumar Dharmalingam Jothinathan, Saantosh Saravanan, Silambarasan Tamil Selvan, Remya Rajan Renuka, Guru Prasad Srinivasan

**Affiliations:** 1 Center for Global Health Research, Saveetha Medical College and Hospital, Saveetha Institute of Medical and Technical Sciences, Chennai, IND

**Keywords:** antibacterial activity, antioxidant activity, toxicity test, senps, green synthesis, anti-angiogenic activity

## Abstract

Introduction

Nanoparticles, owing to their minuscule size, have become pivotal in diverse scientific endeavors, presenting unique characteristics with applications spanning medicine to environmental science. Selenium nanoparticles (SeNPs) exhibit potential in diverse biomedical uses.

Aim

This research investigates the potential anti-inflammatory and anticancer properties of SeNPs, which are synthesized using the green synthesis method. This eco-friendly approach aligns with sustainable practices and utilizes clove extract (*Syzygium aromaticum*).

Materials and methods

Clove extract facilitates SeNP synthesis via sodium selenite reduction. The characterization methods comprised Fourier-transform infrared (FTIR) spectroscopy, UV-VIS spectroscopy, and scanning electron microscopy (SEM). Assessments covered antioxidant properties, chorioallantoic membrane assay (CAM) assay for antiangiogenic effects, toxicity evaluation, and antibacterial assays.

Results

Successful synthesis of SeNPs was verified by a UV-visible absorption peak at 256 nm and FTIR peaks around 3500-500 cm ^-1^, and the spherical morphology was confirmed by SEM analysis with EDAX, which indicated the presence of SeNPs and their unique properties. Phytochemical substances are active chemicals that contribute to the properties of SeNPs. The SeNPs exhibited antioxidant activity with an IC50 value of 0.437 µg/mL and antibacterial properties against bacterial pathogen *Salmonella species*, with a zone of inhibition measuring 19 mm. The CAM assay demonstrated possible antiangiogenic actions, and toxicity testing on *Artemia nauplii* showed biocompatibility.

Conclusion

This study underscores the efficient synthesis of SeNPs using clove extract, emphasizing their potential applications. The notable properties of SeNPs emphasize their promise for diverse biomedical and environmental uses.

## Introduction

Selenium is an essential micronutrient for humans and animals, playing a critical role in the function of selenoproteins [1]. Its nanoparticulate form, selenium nanoparticles (SeNPs), has garnered interest for their potential in biomedical applications due to their unique properties [2].

Clove extract (*Syzygium aromaticum*) represents fragrant dried floral buds sourced from a Myrtaceous family tree, recognized as Lavang in India and known for its rich phytochemical content, has been identified as a potential bio-reducing agent for the green synthesis of metallic nanoparticles, an eco-friendly alternative to conventional chemical synthesis. This study focuses on the synthesis of SeNPs using clove extract, aiming to investigate the anti-inflammatory, anticancer, antioxidant, antibacterial, and anti-angiogenic potentials of SeNPs. Previous research has suggested that SeNPs can mitigate inflammatory damage and induce immunogenic cell death in cancer cells. Our study seeks to expand on these findings, exploring the multifaceted bio-functionalities of SeNPs and their possible applications in biomedical and environmental contexts [3].

Cloves' essential oil also functions as a dental pain reliever and doubles as a carminative by enhancing stomach acidity and aiding peristalsis. Their natural anti-parasitic properties combat intestinal parasites and demonstrate broad antimicrobial effects against fungi and bacteria. Traditionally, cloves have been utilized to treat diarrhea, intestinal worms, and various digestive ailments. Additionally, clove buds function as a biosorbent for heavy metal removal. The primary aromatic compound in cloves is eugenol, constituting 72-90% and possessing substantial anesthetic and antiseptic attributes. Cloves feature a variety of essential constituents, including tannins, gallotannic acid, crategolic acid, vanillin, beta-caryophyllene, acetyl eugenol, and methyl salicylate, recognized for their pain-relieving properties. Additionally, they consist of flavonoids like augmentin, eugenin, kaempferol, and rhamnetin. The triterpenoids present encompass oleanolic acid, stigmasterol, and campesterol, alongside various sesquiterpenes [4].

Clove oil assumes a vital function as the principal reducing agent and stabilizer in the environmentally sustainable synthesis of gold nanoparticles (AuNPs) [5]. Utilizing a green approach, zinc oxide nanoparticles with photocatalytic, antimicrobial, and antioxidant properties are fabricated through hydrothermal synthesis, employing clove hydroalcoholic extract. The process is optimized for enhanced performance [6]. Spyridopoulou et al. investigated the pro-apoptotic activity of SeNPs, revealing their potential to induce immunogenic cell death in colon cancer cells. This study suggests that utilizing SeNPs as a treatment may effectively eliminate tumor cells by inducing apoptotic cell death and stimulating immunological responses [7]. Mediated by cloves, metallic nanoparticles are synthesized, characterized, and explored for potential pharmacological and industrial applications, demonstrating antioxidant, antimicrobial, and anticancer effects [8].

Utilizing a one-step environmentally friendly approach, bimetallic nanoparticles of Au/Ag are synthesized through clove bud extract, leading to improved antioxidant bioefficacy and catalytic activity [9]. Chorioallantoic membrane (CAM) assays, employed to explore angiogenesis, tumor cell invasion, and metastasis, provide distinct advantages. These include the highly vascularized CAM, which enables efficient grafting of tumor cells, ensuring high reproducibility. The method is characterized by its simplicity, cost-effectiveness, and closed system that allows the prolonged examination of limited-quantity anti-metastatic compounds. The CAM, embodying a multilayered epithelium, interacts with the air through the ectoderm, while the mesoderm (stroma) and endoderm interface with the allantoic sac. It also replicates the physiological environment of cancer cells by including various extracellular matrix proteins (ECM) like laminin, fibronectin, integrin α νβ з, and collagen type I [10]. This research aims to proficiently engineer SeNPs employing green synthesis methodologies, utilizing clove extract as a bio-reducing agent. The primary objective is to rigorously assess the multifaceted bio-functionalities of the synthesized SeNPs, encompassing potent anti-inflammatory, anticancer, antioxidant, antibacterial, and anti-angiogenic properties. This comprehensive investigation seeks to elucidate the versatile applications of SeNPs within a spectrum of biomedical and environmental contexts.

## Materials and methods

Collection of materials

The plant *Syzygium aromaticum* was procured from a local vendor, and the sample was authenticated by the Centre for Advanced Studies in Botany at the University of Madras, Chennai, Tamil Nadu, India. The preparation of the clove powder involved a series of steps including washing to eliminate solid particles and dust using double distilled water, followed by a 48-hour drying period at 55°C. The cloves were then finely ground using a mechanical grinder and sifted through a sieve. Subsequently, the resulting biosorbent was preserved for future experimental use.

Aqueous extract preparation from clove

To prepare the clove extract, 25 grams of clove powder was mixed with 500 mL of distilled water and stirred well. The blend was kept overnight, followed by 20-min heating at 60°C. Once cooled to room temperature, it underwent filtration by using Whatman filter paper 110mm and was preserved at 4°C for subsequent experimental analysis [11].

Preparation of sodium selenite solution

To prepare the sodium selenite solution, a standard stock solution (5mM) was made by dissolving sodium selenite (Na_2_SeO_3_) in 100mL of distilled water.

Synthesis of SeNPs

For SeNP synthesis, about 10 mL of *Syzygium aromaticum* aqueous extract (SAAE) was added separately to 90 mL solution of Na_2_SeO_3 _solution (5 mM) and kept stirring at 300rpm at room temperature for 1hr. Then the reaction mixture was incubated at a temperature of 37°C for a duration of 24h. Following the incubation, the color changed from dry brown to pale yellow, which indicated that the SeNPs could be formed due to the redox reaction by phytochemicals present in SAAE with selenium metal ions. This color change was an indication of the synthesis of SeNPs. Control was maintained without the addition of Na_2_SeO_3_, which showed no color changes.

Characterization of SeNPs

Characterization of the SeNPs involved employing scanning electron microscopy (SEM), UV-Vis spectroscopy, and Fourier-transform infrared (FTIR) spectroscopy. Initially, synthesis was identified through UV-visible spectrophotometry (200-800 nm). FTIR analysis (4000-400cm^-1^) identified functional groups on SeNPs. Scanning electron microscopy coupled with energy-dispersive X-ray spectroscopy (EDAX) was employed to assess the morphology and purity [12].

Radical scavenging assay (DPPH)

A solution of 0.1 mM 1,1-diphenyl-2-picryl hydrazyl (DPPH) in methanol was formulated. Mixing extracts at different concentrations (ranging from 2 to 20 μg/mL) with 2 mL of the DPPH solution and an equal volume of distilled water occurred. After being vortexed, the mixture underwent a 30-min incubation in darkness at room temperature, and its absorbance at 517 nm was measured using a spectrophotometer with ascorbic acid as the comparison. The calculation of the DPPH radical scavenging activity percentage was then performed employing the equation:

Radical scavenging activity (DPPH) % = {(A_0_− A_1_)/A_0_} × 100.

The measurements of absorbance for both the control (A_0_) and the extractives/standard (A_1_) were conducted, and the inhibition (%) was graphed against concentration. The determination of IC50 was made based on the plotted data, and this procedure was replicated three times at each concentration to enhance accuracy [13].

Antiangiogenesis activity

The CAM assay was conducted under aseptic conditions in a cell culture facility to ensure a sterile environment. The eggs underwent cleansing using alcohol to prevent contamination. Experimental procedures were executed within a laminar flow cabinet for aseptic conditions, and the equipment was sanitized with 70% alcohol. Three-day-specific pathogen-free embryonated eggs were transported in husks, and embryo presence and location were confirmed using a torch light. The eggs were incubated with no rotational movement applied at a temperature of 37°C and a humidity level of 50%. SeNPs synthesized with SAAE (0.5 mg) were introduced into the eggshell above the air sac, covered, and then underwent a 96-hour incubation at 37°C with 50% humidity. A replicated test was conducted [14].

Artemia franciscana (brine shrimp) toxicity assay

The toxicity of different nanomaterials was evaluated through the brine shrimp lethality bioassay [15]. The commercially available, Artemia cysts (Supreme plus - Golden West Artemia) (*Artimia franciscana*) were used for this study. For the toxicity assessment, a positive control (0.5 mL DMSO in 5 mL seawater), a negative control (1 mg/mL of each nanomaterial dissolved in DMSO), and a standard control (5 mL seawater) were utilized. The negative control was meticulously prepared in triplicate with different concentrations (100, 200, 300, 400, 500 µg/mL) by undergoing serial dilutions, all adjusted to a final volume of 5 mL using seawater.

Recently emerged nauplii of *Artemia franciscana*, ten larvae each, were transferred into the normal and positive controls, as well as vials containing various concentrations of the negative control. Nauplii counts were conducted under a magnifying lens for precise transfer. Vials were kept under light, and the count of surviving nauplii was conducted 24 hours later. The percentage of mortality was determined for every dosage level and the corresponding control using the methodology outlined by Miller and Tainter [16].

Antibacterial activity

The effectiveness of SeNPs, synthesized using SAAE, was evaluated against both Gram-negative bacteria (*Salmonella species, Pseudomonas aeruginosa*) and Gram-positive bacteria (*Staphylococcus aureus, Bacillus subtilis*) for antibacterial activity. The assessment of antibacterial activity utilized the agar well diffusion method, wherein sterilized Petri plates containing 30 mL of Mueller Hinton agar medium were employed. Wells, designated as A, B, C, and D each with a diameter of 6 mm, were created for the evaluation process. 40 µL each of biosynthesized SeNPs and Na_2_SeO_3 _(5 mM) were dispensed into wells A and B, respectively. Well C was filled with 40 µL of Gentamycin (10 µg), and 40 µL of SAAE was introduced into well D. The mean zone of inhibition (ZOI; mm) was measured in mm for all surrounding wells after a 24 h incubation at 37°C.

## Results

Visual observation and UV-visible spectroscopy and FTIR spectroscopy analysis

**Figure 1:**
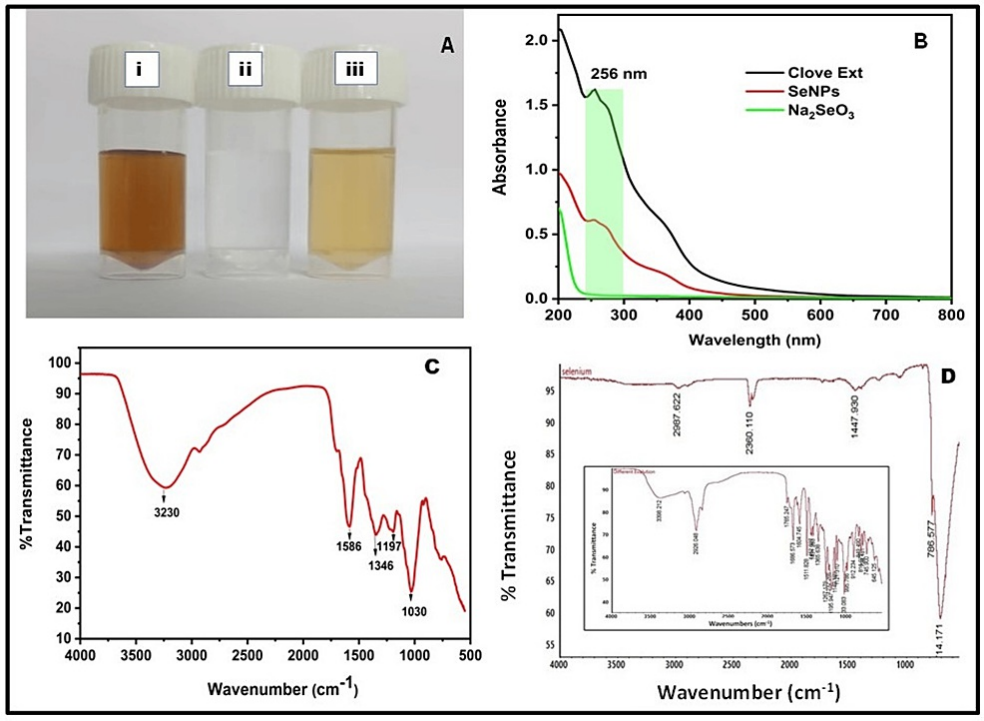
Results of synthesized SeNPs Synthesized SeNPs by using *S. aromaticum* (A) *S. aromaticum* Aqueous Extract (A(i)), Sodium Selenite Solution (A(ii)) and Synthesised SeNPs (A(iii)), (B) UV–Visible spectrum of synthesized SeNPs by using *S. aromaticum* extract, (C) FTIR spectra of synthesized SeNPs by using *S. aromaticum* aqueous extract and (D) FTIR spectra of Sodium Selenite. (D) Inset: *S. aromaticum *extract

The bioreduction of Na_2_SeO_3 _to SeNPs was synthesized by* Syzygium-aromaticum *aqueous extract (SAAE) by color changes of NP solution from dark brown to pale yellow (Figure 1A). The development of a pale-yellow color was observed for all treatments with incubation prolongation, no additional color changes were observed after 4h of incubation at 25° C. SeNPs were synthesized through a simple and environmentally friendly method. SAAE played a dual role, serving as the reducing agent and the stabilizing agent during the synthesis. A clear Na_2_SeO_3 _solution (Figure 1A) was kept as control without adding SAAE. Prior studies have demonstrated the ability of polyphenols in SAAE to reduce Se^4+^ ions and generate metallic SeNPs. Upon adding 5 mM Na_2_SeO_3_ to SAAE, a noticeable color change from dark brown to pale yellow occurred (Figure 1A). This was corroborated by the gradual increase in the optical phenomenon within the characteristic peak (200-400 nm) with an extended reaction time. Unlike Se^4+^ ions, which lack an absorption peak in UV-vis spectra, the UV-visible spectrum of SeNPs (Figure 1B) exhibited a maximum absorption peak at 256 nm. The strong absorption is associated with the SeNPs with an internal excited state [17]. The FTIR spectrum of SeNPs was utilized to characterize the surface functional groups for identification purposes (Figure 1C), revealing notable absorbance bands at specific wavenumbers. The band at 3230 cm^-1^ indicated stretching of O-H intramolecular interactions, the peak at 1586 cm^−1^ was attributed to the stretching of C-C bonds within the aromatic ring. Additionally, a strong peak at 1346 cm^−1^ indicated the stretching of S=O of compounds, and 1194 cm^−1^ represented C-O (-CH2X) in the carbonyl group. The intense peaks observed at ~ 1200 cm^−1^ and 1100 cm^−1^ were ascribed to the vibrational expansion of a carboxyl group (C=O), while 1030 cm^−1^ indicated stretching of C-N in amines with aliphatic (non-aromatic) carbon chains. As shown in Figure 1D, the band at 2987 cm^-1^ indicated O-H stretching, N-H stretching, and C-H stretching groups with alkane, amine salt, alcohol, and carboxylic acid compound classes. 2360 cm^-1^ has O=C=O stretching, and 1447 cm^-1^ has C-H bending with alkane compound classes. The FTIR analysis suggested the involvement of various compounds in capping the synthesized SeNPs, with potential contributions to antibacterial and antioxidant activities. The SAAE FTIR spectrum absorption peak is shown in Figure 1D. Herein, peaks observed at 3398, 2926, 1765, and 1686 cm^-1^ represent several functional groups as N-H, O-H, C-H, C=O, and C=N stretching which indicate that aromatic compound, amines, alcohols, alkanes, and carboxylic acid act as reducing and stabilizing agents for SeNPs.

SEM with EDAX and DPPH assay

**Figure 2:**
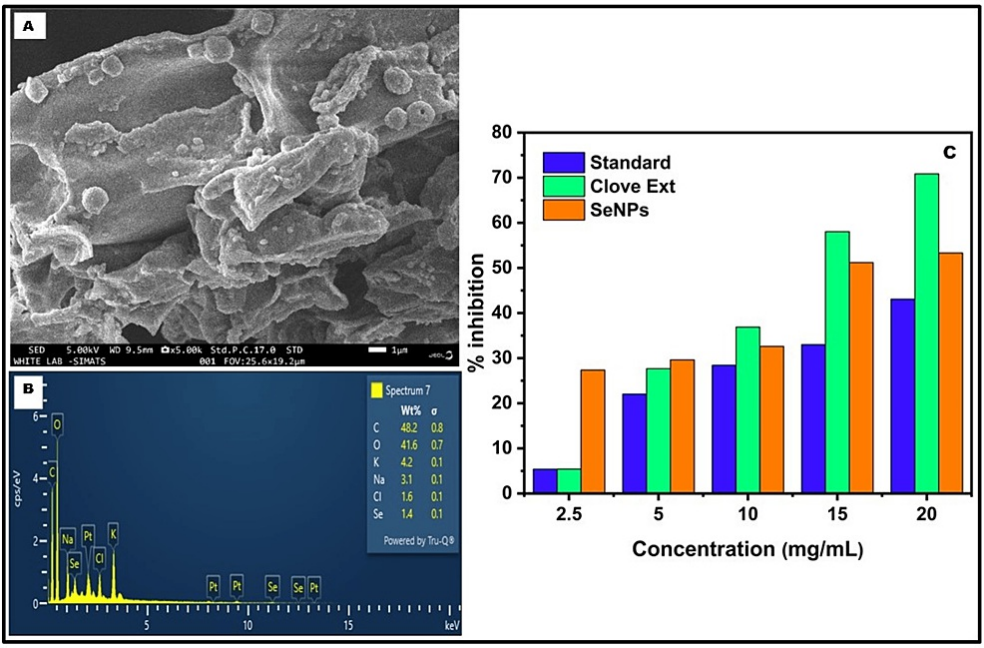
Synthesis of SeNPs (A) SEM images of SeNPs, (B) EDAX of SeNPs synthesized by the *S. aromaticum* extract, and (C) antioxidant assay using DPPH of SeNPs synthesized by *S. aromaticum* aqueous extract.

SEM with EDAX was employed for a morphological study of green-synthesized SeNPs. The SEM images depicted predominantly spherical SeNPs (Figure 2A). EDAX spectra (Figure 2B) of the green-synthesized SeNPs revealed a composition of 1.4% selenium, 42.8% carbon, 41.6% oxygen, and 3.1% sodium. The presence of selenium is associated with the organic matter enveloping the SeNPs, leading to the identification of oxygen and carbon compositions presented in the synthesized SeNPs. The presence of sodium can be attributed to the introduction of NaOH for pH regulation. In Figure 2C, the radical-scavenging potential of SeNPs synthesized using SAAE was demonstrated through the DPPH method. The procedure hinges on the DPPH reduction facilitated by an antioxidant substance, functioning as a supplier of hydrogen. The research evaluated how the SeNPs could inhibit the activity of free radicals, with IC50 values observed around 0.437 µg/mL, while ascorbic acid served as a positive control.

Antiangiogenesis activity assessed by CAM assay

Angiogenesis imbalance, involving pro- and anti-angiogenic molecules, contributes to various pathologies. Cancer, marked by uncontrolled cell growth, relies on tumor angiogenesis for progression and metastasis. SeNPs synthesized with SAAE were tested on the CAM, demonstrating anti-angiogenic effects at 0.5 mg concentration. Results show reduced angiogenesis in chicken embryo blood vessels, analyzed based on thickness, branching, sprouting, and manual vessel counting.

Toxicity test

*Artemia nauplii*, chosen as a model organism for in vivo studies, offers advantages due to the accessibility of artemia cysts, ease of hatching, and simple maintenance in laboratory settings. Additionally, Artemia is considered an ideal crustacean for acute toxicity testing, aligning with the guidelines of the US Environmental Protection Agency. Furthermore, the biological characteristics of Artemia closely resemble those of shrimp larvae, justifying its selection as a model organism to assess the SeNPs' toxicity. The study examined the toxic effects of the "Artemia Reference Center"-test (ARC-test) of SeNPs at varying concentrations. The freshly hatched *Artemia nauplii *were treated with various concentrations (100, 200, 300, 400, and 500 µg/mL) of SeNPs, and the percentage of the mortality rate was indicated in Table 1. Treatment with SeNPs at a concentration of 500 µg/mL led to a 20% mortality rate in contrast with the control group (no mortality recorded). The result showed that the SeNps were not acutely toxic to *Artemia nauplii *even at a higher level (500 μg/mL). The results of the present study imply the biocompatible and safe nature of SeNPs, supporting their potential for various biological applications. For this study, at 500 μg/mL concentration, the nauplii exhibited typical appearance and behavior and there is no mortality was observed up to 300 µg/mL concentration. Hence, the present study clearly demonstrates that the biocompatibility of nanomaterials didn’t cause any significant toxicity in Artemia nauplii within up to 500 μg/mL concentration compared to control.

**Table 1:** Toxicity test results

Conc. (µg/mL)	Total No. of Brine Shrimp	Treatment		% of Mortality	Probits
		Death	Live		
100	10	0	10	0	-
200	10	0	10	0	-
300	10	0	10	0	-
400	10	1	9	10	3.72
500	10	2	8	20	4.16

Antibacterial activity

This study presents the antibacterial activities of SeNPs synthesized from SAAE, represented in Table 2 by the zone of inhibitions (ZOIs). SeNPs from the SAAE exhibited no ZOIs for Na_2_SeO_3 _concentration. The highest antibacterial activity was observed against the bacterial pathogen *Salmonella species*, with a ZOI measuring 19 mm. Additional characterization tests were performed on this sample of SeNPs, and the outcomes underwent statistical analysis. The findings confirmed that SeNPs synthesized from SAAE exhibited antibacterial activity comparable to the standard Gentamycin.

**Table 2:** Antibacterial activity of SeNPs expressed as inhibition zone diameters (mm)

S. NO	Bacteria	Zone of Inhibition			
		Clove Extract	Na_2_SeO_3_	SeNPs	Control
1	Salmonella species	16mm	-	19mm	14mm
2	Staphylococcus aureus	19mm	-	16mm	19mm
3	Bacillus subtilis	16mm	-	17mm	16mm
4	Pseudomonas aeruginosa	10mm	-	12mm	13mm

## Discussion

The primary study was to synthesize SeNPs from SAAE and subsequently examine their potential as antibacterial agents, antioxidants, and anti-angiogenesis agents, and find out their toxicity. *Syzygium aromaticum *is utilized in the production of SeNPs to evaluate potential uses and identify the most effective application. *S. aromaticum* is a medicinal plant with pharmacokinetic and drug-like properties found in its phytochemical composition. Compared to many Western countries, India has a relatively high prevalence of cancer in the modern period. Antibiotics and other medications can treat infections and cancer in certain situations. Still, frequent use of the medication causes the cancer to worsen and become resistant to bacteria during these treatments. The current requirement is the development of different antibacterial, anti-angiogenesis, and antioxidant medications with non-toxic effects.

Although we synthesized SeNPs using an aqueous extract, several researchers have used solvent extract of *S. aromaticum*. The reduction of sodium selenite into SeNPs was subsequently verified using a UV-Vis spectrophotometer measurement. The UV-Vis spectrum's maximum absorbance peaks at 266 and 380 nm offered proof that producing SeNPs using *Luffa cylindrica* in an environmentally friendly manner is feasible. When compared to our results, the absorbance is mediated [18].

FTIR spectroscopy of the synthesized SeNPs was then used to assess the functional groups involved in the reduction of Na_2_SeO_3 _into SeNPs. Our FTIR results were compared to previous studies, and our spectral analysis revealed peaks that were consistent with prior studies, indicating the presence of O-H groups. This shows that the spherical form of the SeNPs is caused by hydrogen bonding between selenium and hydroxyl groups from SAAE. This observation verifies that the synthesis was successful and that the nanoparticles have a biological coating [19,20]. To ascertain the nanoparticle presence and morphology of the SeNPs, SEM with EDAX is utilized for characterization. The mild SeNPs' EDAX results were in line with earlier research findings. Selenium was the primary ingredient, with trace levels derived from SAAE. Synthesis indicates that optimization prospects point to the possibility of higher purity and consistency. The primary objective of subsequent research should be to improve synthesis for particular applications [21,22].

The production of the antioxidant action of SeNPs by SAAE served as a reducing power, which was demonstrated in this study. When compared to normal ascorbic acid (70%), SeNPs made utilizing the SAAE demonstrated a 53% scavenging of free radicals at 20 µg/mL concentrations in the DPPH assay. Based on existing literature, clove extract exhibits significant antioxidant activity, achieving up to 98.6% free-radical scavenging efficiency at 800 μg/mL. This indicates a strong antioxidant potential in clove extract. SeNPs synthesized from other plant sources have demonstrated moderate antioxidant activity. These findings align with previous studies on antioxidant activities, suggesting that while SAAE possesses antioxidant capabilities, SeNPs exhibit superior free-radical scavenging effectiveness, particularly at higher concentrations.

Using chick embryonated eggs and the CAM assay, the earlier analysis discovered that adding SeNPs reduced angiogenesis by over 30% while combining three metal nanoparticles. Significant anti-angiogenic effects linked to the selenium-gold-chitosan (Se-Au-CS) were found using the CAM assay. This is the assessment of Se-Au-CS's strong anti-angiogenesis capabilities using the CAM assay, indicating its possible use in the treatment of a variety of cancer disorders [23]. In our study, we utilized single SeNPs synthesized by SAAE for anti-angiogenesis, showing superior efficacy in the CAM assay compared to the above studies that employed dual nanoparticles for similar effects.

Additionally, SeNPs showed no cytotoxicity on *Artemia salina* larvae and great in vivo survival rates up to 100 μg/mL in previous investigations. This suggests that SeNPs are a viable way to deal with aquaculture's biofilm problems and they might be used in both the cosmetic and medicinal industries [24,25].

The recent focus on nanoparticles for disease control is due to their remarkable antibacterial properties, with SeNPs standing out for their potent activity. Essential oils from *S. aromaticum* are effective against pathogens like *Escherichia coli *and *Staphylococcus aureus*, challenging antibiotics such as gentamicin [26]. However, SeNPs have demonstrated superior antibacterial activity across a wider range of bacteria, including *Mycobacterium tuberculosis* and *Pseudomonas aeruginosa*, outperforming SAAE. This highlights the difference in efficacy between plant extracts and metal nanoparticles in bacterial inhibition. The high affinity of SeNPs for bacterial cell membranes may explain their bactericidal action, although the exact mechanism remains unclear. Unlike plant extracts that rely on a variety of natural antibacterial agents, SeNPs utilize their small size and large surface area for effective bacterial interaction, enhancing their antibacterial effects. This differential efficacy underscores the advanced antibacterial potential of nanoparticles, offering unique advantages in various application contexts [27-29].

Overall, the study shows promising results for the synthesis and potential applications of SeNPs using SAAE, in vivo studies, biocompatibility, stability, and comparative analysis through further research and experimentation would strengthen the findings.

Limitations

The current research investigated the cytotoxic, antibacterial, antiangiogenic, and antioxidant characteristics of SeNPs using in vitro analyses, such as the DPPH assay. We also examined their potential for reducing inflammation, treating diabetes, and fighting cancer. SeNPs have shown potential for many biological uses, underscoring the importance of pinpointing their active elements for a more comprehensive comprehension. Yet, the dependence on in vitro tests restricts the relevance of our results. Future research should prioritize in vivo studies, including animal experiments and clinical trials, to provide a thorough understanding of the effects, safety, and practical use of SeNPs, in order to address the existing limitations of our work.

## Conclusions

This study successfully synthesized SeNPs using SAAE, showcasing a green, cost-effective synthesis method. The color change and FTIR analysis confirmed the synthesis and organic compound incorporation from SAAE. SeNPs demonstrated significant antibacterial activity, particularly against Salmonella species, by disrupting cellular functions and metabolic processes. Toxicity studies revealed that high concentrations of SeNPs adversely affect *Artemia nauplii*, indicating a need for dosage control. Additionally, SeNPs exhibited antiangiogenic properties, suggesting potential in cancer therapy by inhibiting tumor growth. These findings highlight SeNPs' versatility and promise for future biomedical applications, underscoring SAAE's role in producing biocompatible nanomaterials.
